# Supplementary material for: Effect of the Resveratrol Rice DJ526 on Longevity
Source: Nutrients. 2019 Aug 5;11(8):1804. doi: 10.3390/nu11081804 (PMC6723356; doi:10.3390/nu11081804)
Supplement: Supplementary file 1 [file nutrients-11-01804-s001.pdf]

Supplementary Materials

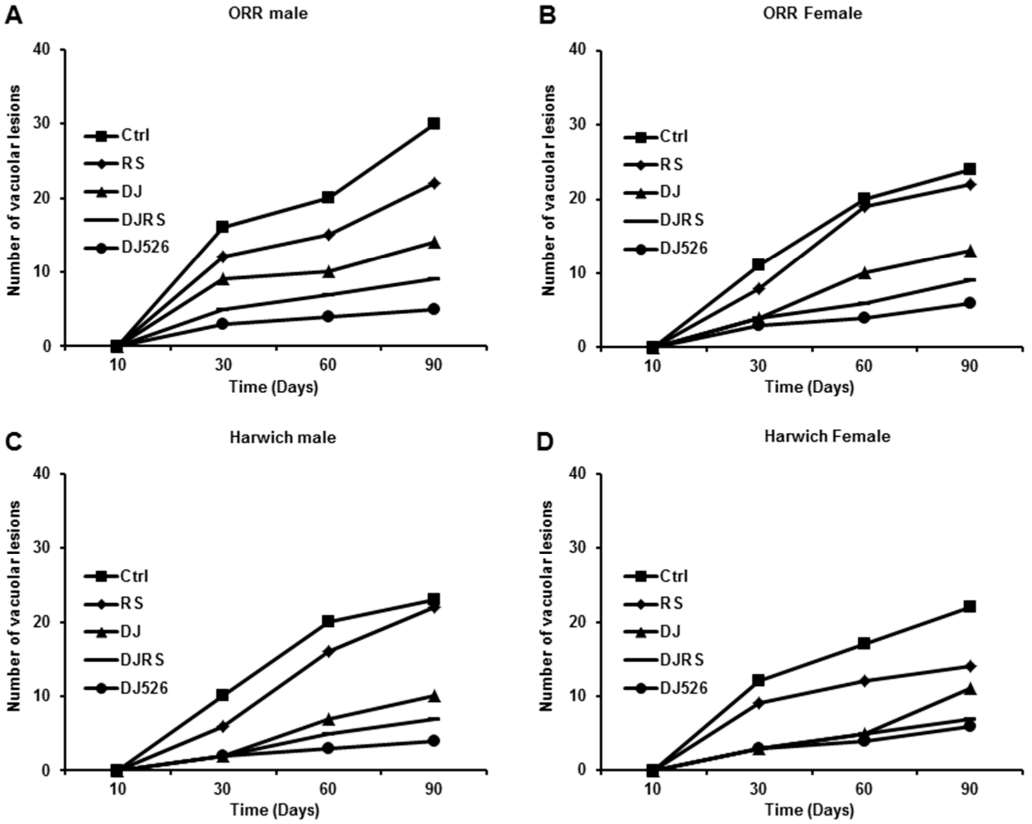

Figure S1. The resveratrol rice DJ526 reduced the number of vacuolar lesions in the brains of *D. melanogaster*. **(A-D)** The number of age-related vacuolar lesions in the brains of male and female flies from the ORR and Harwich strains. The Ctrl represents standard cornmeal medium; RS represents cornmeal medium supplemented with resveratrol at 31.54 μg/L, the equivalent amount of resveratrol found in the DJ526 media; DJ represents the medium in which 50% of cornmeal was replaced with Dongjin rice; DJRS represents the medium in which 50% of cornmeal was replaced with Dongjin rice and supplemented with resveratrol at 31.54 μg/L, and DJ526 represents the medium in which 50% of the cornmeal was replaced with the resveratrol rice DJ526 (Table S1). The quantification of the vacuolar lesions based on the histological analysis of the *Drosophila* brains were observed at the 10th, 30th, 60th and 90th days post-eclosion (n=30).

Table S1. Media compositions used to maintain the *D. melanogaster* ORR and Harwich strains.

Ingredient	Ctrl	RS	DJ	DJRS	DJ526
Corn meal (g/L)	84	83.99	42	42	42
Active dry yeast (g/L)	24	24	24	24	24
Sucrose (g/L)	47	47	47	47	47
Agar (g/L)	8	8	8	8	8
Molasses (ml/L)	25	25	25	25	25
10% Methyl perahydroxybenzoate (ml/L)	10	10	10	10	10
Propionic acid (ml/L)	4	4	4	4	4
Resveratrol (μg/L)	0	31.54	0	31.54	0
DJ rice (g/L)	0	0	42	42	0
DJ526 rice (g/L)	0	0	0	0	42
